# The Knowledge Level and Opinions of Physicians about the Medical and Legal Procedures Related to Physical Child Abuse

**DOI:** 10.4274/balkanmedj.2015.1195

**Published:** 2017-03-28

**Authors:** Sema Demirçin, Akın Tütüncüler, Fatmagül Aslan, Sevtap Velipaşaoğlu Güney, Mehmet Atılgan, Hakan Gülkesen

**Affiliations:** 1 Department of Forensic Medicine, Akdeniz University School of Medicine, Antalya, Turkey; 2 Office of Forensic Medicine Council, İstanbul, Turkey; 3 Clinic of Forensic Medicine, Antalya State Hospital, Antalya, Turkey; 4 Department of Pediatrics, Akdeniz University School of Medicine, Antalya, Turkey; 5 Department of Biostatistics, Akdeniz University School of Medicine, Antalya, Turkey

**Keywords:** Child abuse, physician, postgraduate, training, questionnaire

## Abstract

**Background:**

In order to diagnose child abuse, physicians need to consider the possibility of abuse in every child they encounter, have sufficient information about the topic and manage the cases according to current law.

**Aims:**

To determine the knowledge level of physicians on child abuse and to learn their opinions about the procedures when they suspect child abuse.

**Study Design:**

A questionnaire (cross-sectional) study.

**Methods:**

A detailed questionnaire was applied to 390 physicians of whom 233 were general practitioners. The first part of the questionnaire included demographic variables (age, gender, occupational experience) and the frequency of child physical abuse cases encountered, since that is the most easily diagnosed and proven form of abuse. The second part consisted of 32 questions about diagnosis of physical child abuse and procedures during the follow-up of the cases. Statistical analyses were performed using SPSS version 18.0.

**Results:**

Of the participating physicians, 47.4% (n=185) were female and only 13.1% of the physicians had some kind of postgraduate training on child abuse. The correct response rate of specialists compared to general practitioners was significantly higher. A total of 263 (72.3%) physicians thought that there was a specific law on physical child abuse in the Turkish Republic. More than two-thirds of physicians thought that reporting should only be addressed to Social Services and physicians should not be obliged to report to law enforcement.

**Conclusion:**

The results of the present study adds to the already known necessity for better training of physicians about physical child abuse and the need to refresh their knowledge through postgraduate courses. According to current regulations, it is obligatory to report abuse cases to the public prosecutor and/or police, therefore physicians also need training in respect of the legal status and medico-legal approach to these cases.

Child abuse can be seen in all societies. In a study of 1093 high-school students, Schilling et al. (1) revealed that 5% of females and 8.6% of males were physically abused during their childhood, while Chapman et al. (2) showed that of 9460 study participants, 1456 males and 1394 females had been subjected to physical child abuse. In the USA, three million child abuse cases per year are reported. Statistics from the USA for the period 1995-1997 show that 41% of deaths due to child abuse had been previously admitted to child protection services (3).

There is no published report representing the whole of the Turkish Republic on this topic. Sofuoğlu et al. (4) reviewed the experiences of violence in 7540 students, in three provinces (İzmir, Zonguldak and Denizli) of the Turkish Republic and the frequency of physical abuse was determined as 58.1% in participants in the period of February-May, 2012. The Research Study on Child Abuse and Domestic Violence in the Turkish Republic conducted by the Ministry of Social Services and Child Protection Agency reported a rate of physical abuse of 45% in 235 children living in six provinces (İstanbul, Samsun, Konya, Şanlıurfa, Adana and Erzurum), in 2008 (from January 24 to March 23) (5). The results of these studies reveal that half of the participating children had experienced physical abuse and that it is a common problem in Turkish society.

The duties of physicians in respect of child abuse can be regarded as keeping the probability of this diagnosis in mind for every child they encounter, having current knowledge about the topic and managing the cases according to the cultural and legal norms of the country (1). In the Turkish Republic, as in many other countries, general practitioners, family physicians or paediatricians are the first to examine a child. Undergraduate courses in medical schools are usually regarded to be sufficient to manage these cases and generally no further training is provided during the postgraduate period.

In this study, the aim was to determine the knowledge of physical child abuse of physicians who provide services to children, and to evaluate whether their demographic characteristics or occupational experience had any effect on the diagnosis of child abuse. The views of the physicians about the procedures during the follow-up of these cases were also evaluated.

## MATERIAL AND METHODS

A detailed questionnaire was applied to 390 physicians working in 54 primary care units, 2 state hospitals, 5 private hospitals and 1 university hospital in Antalya. Of the physicians, 233 were general practitioners, and the rest were either specialists or residents in the departments of paediatrics (n=105), emergency medicine (n=29), paediatric surgery (n=16) and family medicine (n=7). Ethics committee approval was received for this study from the Akdeniz University School of Medicine. Permission for the study was obtained from Antalya Chamber of the Turkish Medical Association and Provincial Directorate of Health. Informed consent was obtained from the participants.

The questionnaire presented to the participants comprised two parts. The first part included questions about demographic characteristics (age, gender, occupational experience, training on child abuse) and the frequency of encountering child physical abuse cases, since that is the most easily diagnosed and proven form of abuse. The second part consisted of a questionnaire with 32 items developed and validated by Açık et al. (6), including questions about their knowledge on physical child abuse and their opinion about the procedures during the follow-up of these cases.

### Statistical analyses

Statistical analyses were performed using Statistical Package for Social Sciences (SPSS) version 18.0 (SPSS Inc., Chicago, Illinois, USA). The chi-square test was used to compare nominal variables and the Shapiro-Wilk test to evaluate normality. The groups were compared using the Kruskal-Wallis test, then the groups were compared with each other using the Mann-Whitney U test. Bonferroni correction was used for pairwise comparisons.

## RESULTS

Of 416 general practitioners working in different health centres, 259 (62.3%) could be contacted and of these, 233 (90.0%) agreed to participate in the study. Those who did not complete the questionnaire (n=26, 10.0%) reported time constraints or dislike of their knowledge being tested as the main reasons for unwillingness to participate in the study.

Of the 39 specialists working in the State Hospital, 28 (71.8%) could be contacted and 23 (82.1%) completed the questionnaire. In the university hospital, 110 (90.1%) of 122 residents and fellows participated in the study. From the private hospitals 19 paediatricians, 2 family specialists and 1 paediatric surgeon completed the questionnaire.

Of the 390 physicians, 351 (90%) were working in the public sector and 39 (10%) in the private sector. Overall, 233 (59.8%) of the participants were general practitioners, 71 (18.2%) were paediatricians, 22 (5.6%) were emergency medicine residents, 34 (8.7%) were paediatrics residents, 7 (1.8%) were emergency medicine specialists, 9 (2.3%) were paediatric surgeons, 7 (1.8%) were paediatric surgery residents, 6 (1.5%) were family medicine specialists and 1 (0.3%) was a family medicine resident.

Nearly half of the respondents were male (n=205, 52.6%). The median age was 38 years (range, 24-64 years), and the mean working years as a physician was 13.6 (range, 1-41 years) (Table 1).

No reply was given to the question about encountering a physical child abuse case during their professional life by 25 (6.4%) of the participants. The frequency of encountering physical child abuse victims was ‘a few each year’ in 49.9% (n=182), ‘a few each month’ in 12.0% (n=44), ‘a few each week’ in 2.5% (n=9), and ‘a few each day’ in 2.2% (n=8). While 4.9% (n=18) of the participants stated that they had seen only one case during their professional life and 13.2% (n=48) of participants reported ‘less than one case a year’, 15.3% (n=56) had not encountered any physically abused children.

Only 51 of the participants (13.1%) had received any kind of postgraduate training on child abuse, while 339 (86.9%) had not.

The response rate to the questions developed by Açık et al. (6) to assess the knowledge of child abuse was between 90.5% and 100% (353 and 390, respectively). The rate of correct answers to each item differed between 14.8% and 98.4% (Table 2).

No participant replied correctly to all 11 items presented in the article reported by Açık et al. (6) (marked as an asterisk on Table 2). Only 4 (1.2%) respondents replied correctly to 10 of these 11 items and the rates of correct answers to each item differed between 14.8% and 91.3%.

The overall correct response rates did not differ according to the occupational experience, age or gender of the participants. However, the correct response rate of female physicians to items 1 (p=0.020) and 13 (p=0.009) was higher than that of males and the correct response rate of male physicians to item 12 (p=0.027) was higher than that of females.

The mean number of questions correctly answered by general practitioners was 17.6 (range 7-24, standard error 0.2), by residents 18.4 (range 12-23, standard error 0.4) and by specialists 18.5 (range 11-24, standard error 0.3) (p=0.011). The correct response rate of specialists compared to general practitioners was significantly higher (p=0.012).

The response to the question ‘what makes you think the child is at high risk of abuse?’ was given as ‘anxious or worried child’ by 305 (78.2%) physicians, ‘dirty or uncared-for clothing’ by 55 (14.0%), ‘child in a crying fit’ by 24 (6.2%), ‘rural child’ by 3 (0.8%), and no response was given by 3 (0.8%). The physicians thought that the child’s emotional status was more informative than clothes and personal hygiene (Table 3).

Participants were asked what would be the ideal legal system to report suspected child abuse cases. A very small number (0.8%) stated that ‘reporting would breach confidentiality’, 1.1% thought that ‘abuse should be reported only by the private physician of the child’, and 6.8% wanted ‘physicians to be exempted from the legal procedures resulting from reporting’. While 22.1% were willing to ‘report the suspected cases primarily to Social Services’, 69.2% stated that ‘reporting should only be addressed to Social Services and physicians should not be mandated to report to law enforcement’ (Table 3).

Strategic approaches to prevent child abuse in the Turkish Republic were stated as ‘eliminate poverty’, ‘reduce adolescent pregnancy’, ‘parental education in compulsory schooling’, and ‘well-established family planning implementation’ by 93.6% (n=364) of physicians, with 1 physician stating only the first option, 2 physicians stating only the second, 18 only the third and 4 only the fourth (Table 3).

A total of 263 (72.3%) physicians thought that there was a distinct law on physical child abuse in the Turkish Republic, 101 (27.7%) physicians thought there was not and 26 did not respond to the question (Table 3).

Reasons for hesitating to report physical child abuse cases were: not having sufficient information about family dynamics (1.6%), inadequate training about the topic (6.8%), regarding child abuse as a social issue rather than a medical problem (6.5%), avoiding the emotional burden of the situation since there is no effective system to help these children (9.9%) and a combination of all these factors (75.2%) (Table 3).

## DISCUSSION

The responses of the physicians suggest that encountering child abuse victims is quite prevalent in Antalya. A total of 84.7% of the physicians reported that they had encountered physical child abuse victims at least once in their occupational life.

Most of the physicians (75.7%) stated that Social Services should immediately be contacted by phone and a written report should then be prepared whenever there is suspected child abuse. Almost the same response rate (78.8%) was observed for this item in a study by Açık et al. (6). This demonstrates that most physicians know when to start reporting. However, all of the participants stated that they hesitated to report physical child abuse cases. Their reasons for hesitating to report were not having sufficient information about family dynamics (1.6%), inadequate training about the topic (6.8%), regarding child abuse as a social issue rather than a medical problem (6.5%), avoiding the emotional burden of the situation since there is no effective system to help these children (9.9%) and a combination of all these factors (75.2%). There was no significant correlation between specialties and the above-mentioned factors causing hesitation.

The reluctance of physicians to report has been documented in previously published reports (6,7). In a study in Israel, 43% of paediatricians and family physicians stated that they were not comfortable with reporting physical child abuse cases. It has also been shown that characteristics of physicians such as specialty, level of knowledge, gender or age do not have any effect on attitudes towards reporting (8). Studies from Kuwait, Finland, Sweden and the United Kingdom also reveal similar results (9,10,11,12). A study from the United Kingdom demonstrated that the most common reason for not reporting was the failure to diagnose child abuse. It was shown that infants with suspected inflicted subdural haemorrhages were not thoroughly investigated and thus were not reported to Social Services (11).

In a study in the USA, Theodore and Runyan showed that a significant number of paediatricians were reporting suspected child abuse and 96% had reported a case at least once. However, 30% stated that they may avoid reporting such cases due to the risk of involvement in the court proceedings (13). Van Haeringen et al. (14) revealed that general practitioners were more eager to report than paediatricians. In contrast, Levi and Brown (15) determined no difference between paediatric subspecialties or between paediatricians and other medical specialties in terms of approach to child abuse and reporting.

In the present study, correct answer rates to each item differed between 14.8% and 91.3% for the 11 items marked with an asterisk in Table 2. Using these questions, Açık et al. (6) determined a correct response range of 4% to 93.7% among general practitioners working in four different cities (Elazığ, Malatya, Bingöl, Tunceli) of the Turkish Republic. In the current study, the correct answer rates to each item differed between 14.8% and 98.4% for 27 of 32 items, and the remaining 5 items demonstrated personal opinions, as presented in Table 3. There was a significant difference between the correct response rate and branch in this study. The correct response rate of specialists was significantly higher than that of general practitioners. However, there was no significant difference between residents and specialists and between residents and practitioners. Using three case examples in Denmark, Reijnders et al. (16) demonstrated that 48-91% of general practitioners could identify signs of abuse, but only 15% could diagnose it. Although the first step in the diagnosis of abuse is keeping it in mind as a diagnostic possibility, it should not be forgotten that the level of knowledge of the professionals is crucial for it is management.

In the current study group, there was no statistically significant difference in the correct response rates of general practitioners and residents. This finding suggests that information learned during medical training can be forgotten if not supported by postgraduate training. Furthermore, 86.9% of the participants stated that they had not received any postgraduate training on child abuse.

In a study of 1237 emergency department physicians to evaluate the level of knowledge, attitudes towards and the need for further education on child abuse, Markenson et al. (3) found that only 3% did not want any further training. In Finland, 60% of physicians and nurses working in a university hospital were seen to need further training for the diagnosis of child abuse (10). A London-based study revealed the gap in knowledge on child protection between general practitioners and paediatricians (11). One of the limitations of the current study was that no information was sought on whether or not the participants felt a need for training.

However, in a study by Orhon et al. (17), 40% of parents, 56% of paediatricians and 56.5% of medical school students viewed beating as an acceptable method for disciplining a child. In the same study, burning a child with a cigarette was not seen as a reportable incident by 2.6% of paediatricians and 2.8% of medical school students (17). In societies like Turkey where physical punishment of children is an acceptable or usual discipline method, physicians can be confused about what constitutes child maltreatment and thus fail to report it (18). Even if all legal background is optimally arranged, failure in the recognition and reporting of the cases will continue to hinder the solution. Therefore, refresher training that emphasizes the importance of diagnosing and reporting child abuse is needed during the postgraduate period. In order to attain the attendance of physicians, such training sessions have to be supported and promoted by employers and professional associations.

There are three main findings of this study. The first is that in Antalya, as in different parts of Turkey or other countries, regardless of being a specialist or a general practitioner, a significant number of physicians lack the necessary knowledge to prevent them from missing child abuse cases. Physicians who have a high probability of encountering child abuse victims need to be trained both theoretically and practically through case discussions. Studies have shown that education on child abuse should include the dynamics of family relationships and discussions about the differential diagnosis of accidental and non-accidental trauma (10). The second finding of this study is that physicians fail to report suspected child abuse. The causes of under-reporting have previously been seen to include factors such as lack of knowledge or experience, concerns about facing the families during the trial, worries about the legal procedures and standing in court, and busy working schedules that cannot compensate for time spent in court hearings and accumulation of routine work (8,11,13). Consequently, the third finding is that reporting to Social Services is preferred by the physicians rather than to the Public Prosecutor and police, and that physicians do not want to be involved in the legal procedures of reported cases. This is most probably because in Turkey, independent of the judicial case of abuse, the physician could be immediately accused of malpractice or misconduct by the abuser or other family members as a cover-up of the crime or for retribution.

The most striking result of the study is that a total of 72.3% physicians thought that there was a distinct and specific law on physical child abuse in Turkey and 27.7% of physicians thought that there was not. There is no specific law on physical child abuse in the Judicial Code in Turkey, but only some offences related to physical violence to a person where the penalty is increased when the victim is a child. For example, the sentence is increased from 1-3 years to 2-5 years imprisonment for intentional injury with simple results and a prison sentence is increased one- to twofold for an intentional injury with complicated results.

In a study conducted to evaluate the efficiency of Dutch physicians in describing injuries, although it was not asked, 23.1% stated that it should be the responsibility of forensic medicine specialists to define and evaluate injuries that may result in lawsuits (19). In the current study, 8.7% (n=34) of physicians added notes stating similar opinions. In most of the incidents in Turkey, forensic medicine specialists see the child after the wounds have healed and courts always request a forensic medicine specialist report before making a decision. Therefore, forensic medicine specialists try to identify abuse victims through investigation of their medical records. It is hard, and sometimes even impossible to make comments on a case when the records are not complete, details are missing and patient files are not properly organized. Early consultation with a forensic medicine specialist may eliminate the making of subjective evaluations based on a retrospective review of patient files and may lead to more correct and rapid management of the medico-legal problem. A multidisciplinary approach is required for prevention, diagnosis, management and rehabilitation of child abuse cases and, irrespective of specialty, physicians who examine the child have a key role in resolving this tragic public health problem.

## Figures and Tables

**Table 1:**
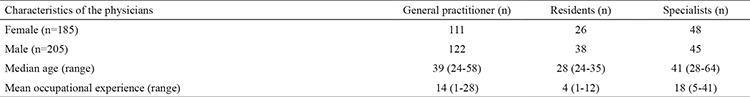
Characteristics of the participating physicians

**Table 2:**
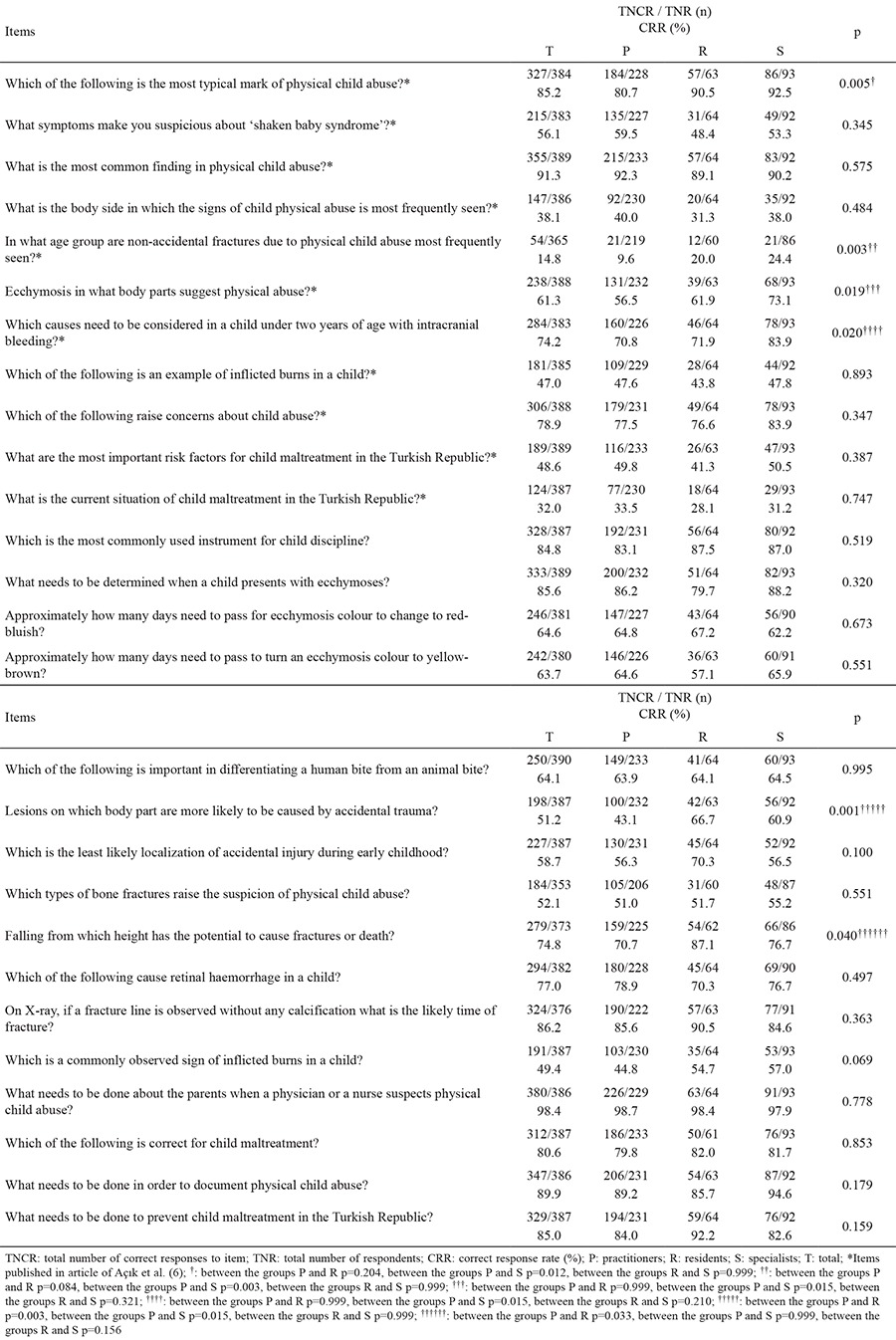
Correct response rate of 390 physicians

**Table 3:**
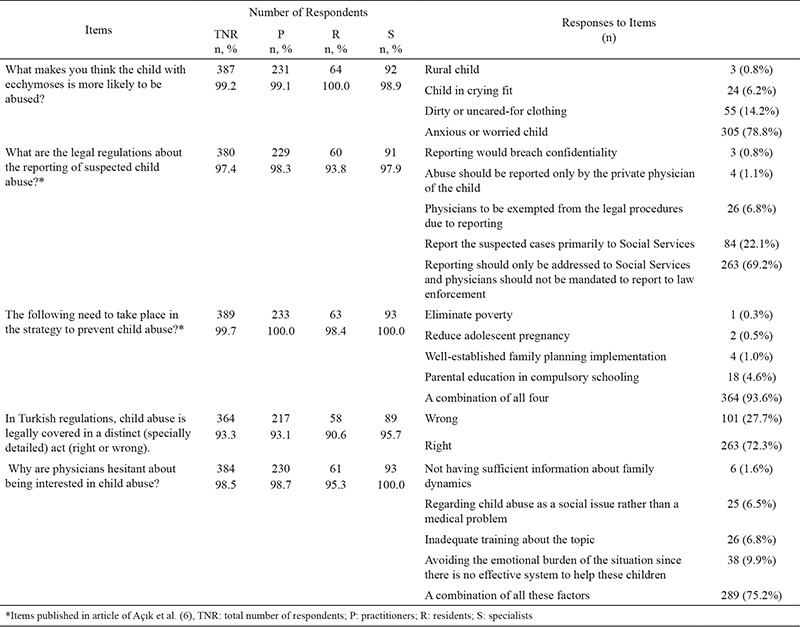
Personal experience and opinions of physicians on child abuse, related to the items
